# Detection of OXA-1 β-Lactamase Gene of *Klebsiella pneumoniae* from Blood Stream Infections (BSI) by Conventional PCR and *In-Silico* Analysis to Understand the Mechanism of OXA Mediated Resistance

**DOI:** 10.1371/journal.pone.0091800

**Published:** 2014-03-19

**Authors:** Madhan Sugumar, K. M. Kumar, Anand Manoharan, Anand Anbarasu, Sudha Ramaiah

**Affiliations:** 1 Prof. Benjamin M. Pulimood Laboratories for Infection, Immunity and Inflammation, Medicine Unit I & Infectious Diseases, Christian Medical College, Vellore, Tamil Nadu, India; 2 Medical & Biological Computing Laboratory, School of Biosciences and Technology, VIT University, Vellore, Tamil Nadu, India; Cornell University, United States of America

## Abstract

*Klebsiella pneumoniae* strains producing extended-spectrum β-lactamases (ESBL) exhibit resistance to antibiotic classes. The production of ESBLs (TEM-1, TEM-2, SHV-1, OXA-1) results in resistance to ampicillin, ticarcillin, piperacillin and cephalosporins. High levels of β-lactamases leads to development of resistance to β-lactamase inhibitors. The present study deals with characterizing antimicrobial resistance pattern among septicemia causing *K. pneumoniae* and the prevalence of inhibitor resistant OXA-1 β-lactamase genes among them. Of 151 study isolates, 59 were resistant to piperacillin/tazobactam and these isolates were further selected for *bla*OXA-1 screening. Amplification of β-lactamases genes by conventional PCR showed the presence of *bla*OXA-1 genes among 12 *K. pneumoniae* (20.3%) isolates. OXA-1 β-lactamase producing strains were found to be resistant to piperacillin/tazobactam(100%), levofloxacin (91.6%), amikacin (75%), cefoxitin (50%), ertapenem (25%), imipenem (16.6%) and meropenem (16.6%); all were susceptible to tigecycline. 3D models of OXA-1 β-lactamase were generated and docking was performed with various β-lactam antibiotics. Molecular docking (MD) revealed the molecular basis of drug sensitivity. MD simulation results clearly confirmed the notable loss in stability for tigecycline-*bla*OXA-1 complex. Findings of the present study will provide useful insights for understanding the mechanism of resistance and help with strategies for the development of new antibiotics. The conventional PCR assay designed in this study can be routinely used in clinical microbiology laboratories to determine the *bla*OXA-1genes.

## Introduction

*Klebsiella pneumoniae* is a Gram negative, non motile, encapsulated, facultative anaerobic, lactose fermenting, rod shaped bacterium. The range of clinical diseases caused by this includes pneumonia, thrombophlebitis, urinary tract infection (UTI), bacteremia and septicemia. Indiscriminate antibiotic use is a major factor that often result in multi drug resistant strains. The production of broad-spectrum β-lactamases (TEM-1, TEM-2, SHV-1, OXA-1) results in resistance to ampicillin, ticarcillin, piperacillin and cephalosporins. Three enzymatic mechanisms have been described for resistance to inhibitor penicillin combinations: i.) production of class C chromosomal β-lactamase [1], ii.) hyperproduction of plasmid-mediated TEM-1and TEM-2 [2], and iii.) production of OXA-1 β-lactamase [3]. The *bla*OXA-1 gene has been found in plasmid and integron locations in a large variety of Gram-negative organisms. The *bla*OXA-1 gene has frequently been found to be associated with genes encoding extended-spectrum β-lactamases (ESBLs). OXA-1 β-lactamase, like most OXAs, hydrolyzes amino and ureidopenicillins (piperacillin) significantly and hydrolyzes narrow-spectrum cephalosporins weakly. In addition, *bla*OXA-1 hydrolyzes broad-spectrum cephalosporins, conferring reduced susceptibility to cefepime and cefpirome. Recent studies have reported very frequent association of *bla*OXA-1 with the worldwide-spread CTX-M-15 ESBL determinant found among human *E.coli* isolates from diverse geographical origins. This association of *bla*OXA-1 with *bla*CTX-M genes makes isolates resistant to β-lactam-β-lactamase inhibitor combinations [4]. Studies conducted by our group over the last 5 years has shown increasing ESBL proportion [5] with multiple co carriage of ESBL, Amp C and NDM enzymes. There are not many Indian studies on *bla*OXA-1 gene, through there are a few studies on *bla*OXA-1 gene along with association of ESBL (TEM, SHV and CTX-M) genes. Therefore, we attempted to evaluate the prevalence of *bla*OXA-1 gene in our settings and determine an *in-silico* approach that explores the mechanism of resistance by OXA-1 β-lactamases.

## Materials and Methods

In this prospective laboratory based surveillance study, Gram-negative strains *K. pneumoniae* (n = 151) determined to be clinically significant from blood stream infections were collected at from five Indian tertiary care centers namely-All India Institute of Medical Sciences (AIIMS)-New Delhi, Amrita Institute of Medical Sciences (AIMS)-Kochi, Sanjay Gandhi Post Graduate Institute of Medical Sciences (SGPGIMS)-Lucknow, Mahatma Gandhi Institute of Medical Sciences (MGIMS)-Wardha, and Jawaharlal Institute of Post Graduate Medical Education & Research (JIPMER)-Puducherry as part of Indian Council of Medical Research (ICMR) sponsored study on clinico-epidemiologic and molecular characterization of extended-spectrum beta-lactamase(ESBL) producing *Klebsiella pneumoniae* (2007–2010). The identities of all strains submitted were reconfirmed by conventional biochemical methods and API (Biomerieux, Craponne, France) system.

The antimicrobial susceptibility test were performed to determine the resistance profile of antimicrobial agent against: ampicillin (10 mg), cefotaxime (30 mg), ceftazidime (30 mg), cefoxitin (30 mg), cefepime(30 mg), cefpirome(30 mg), piperacillin/tazobactam(100 mg/10 mg), amikacin(30 mg), levofloxacin(30 mg), imipenem(10 mg), meropenem(10 mg), ertapenem(10 mg) and tigecycline (10 mg) were determined by the Kirby Bauer disc diffusion method. Piperacillin/tazobactam resistant isolates were used for further screening. Results were interpreted as per the Clinical and Laboratory Standards Institute (CLSI) 2011 guidelines [6].

MIC was performed to determine the resistance profile to the following antimicrobials: ampicillin, cefotaxime, ceftazidime, cefoxitin, cefepime, cefpirome, piperacillin/tazobactam, amikacin, levofloxacin, imipenem, meropenem, ertapenem and tigecycline. E-test (Biomerieux, Craponne, France) method was used and results interpreted as per manufacturers guidelines.

### Molecular Methods

A single colony of each organism was inoculated from a blood agar plate into 5 ml of Nutrient broth (BD, MD, USA) and incubated for 16–18 h at 37°C. Cells from 1.5 ml of the overnight culture were harvested by centrifugation at 8000 rpm for 5 min. After the supernatant was decanted, the pellet resuspended in 500 µl of distilled water. Then cells was lysed by heating at 95°C for 10 min, and cellular debris was removed by centrifugation at 8000 rpm for 5 min. Supernatant, (2 µl), was used as the DNA template source for amplification.

The supernatant (2 µl) was used as the source of DNA template for amplification. PCR was performed with a final volume of 25 µl in 0.2-ml thin-walled tubes. The primer sequences were as follows : *blaOXA-1* forward, 5′ TTTTCTGTTGTTTGGGTTTT 3′; *blaOXA1* reverse, 5′ TTTCTTGGCTTTTATGCTTG 3′ [7]. Each reaction contained 20 mM Tris-HCl (pH 8.4); 50 mM KCl; 0.2 mM each deoxynucleotide triphosphate; 1.5 mM MgCl2; primers OXA-1 F, OXA-1 R, and 1.25 U of *Taq* DNA polymerase (Fermentas). The PCR program consisted of an initial denaturation step at 95°C for 2 min, followed by 30 cycles of DNA denaturation at 94°C for 45 s, primer annealing at 55°C for 30 s and extension 72°C, 1 min [7]. After the last cycle, a final extension step at 72°C for 5 min was added. Fifteen-microliter aliquots of PCR product were analyzed by gel electrophoresis with 2% agarose (USB Corporation, Cleveland, USA). Gels were stained with ethidium bromide at 1.5 µg/ml and visualized by UV transillumination. A 100-bp DNA ladder (Fermentas International Inc. Burlington, Canada) was used. Clinical strain 3MBO9 (*bla*OXA-1, 427 bp) was used as positive control, negative control was PCR mix with water in place of template DNA.

The statistical analysis was conducted using SPSS package version 16.0 (IBM, New York, USA). Pearson product moment correlation was used to test for the association between variables (ESBL production and antibiotic resistance).The statistical analysis was run at 95% confidence limit and p- values<0.006 were considered as significant.

### Bioinformatics

The Target sequence of *bla*OXA-1 was retrieved from UniProt [8], accession number: R4WC18 and modelled using modeller [9].

Constructed 3D-model of *bla*OXA-1 was used for refinement and model quality validation. Energy minimization and refinement of predicted 3D model of *bla*OXA-1 was performed in swiss PDB viewer [10]. The refinement of the final model was carried out through energy minimization using 1000 iterations of steepest descent (SD) calculation to remove bad contacts between protein atoms. PROCHECK [11] was used for model quality evaluation.

After elucidation of final 3D model, the possible binding site of *bla*OXA-1 was searched using Q-siteFinder [12]. Q-siteFinder recognized ten binding sites for *bla*OXA-1 using van der Waal's probes and interaction energy. A set of 13 drugs were used for this docking study. The coordinates of ampicillin [CID 6249], cefotaxime [CID 57442673], ceftazidime [CID481173], amikacin [CID:37768], levofloxacin [CID:149096], imipenem [CID:104838], meropenem [CID:441130], cefepime [CID:5479537], cefpirome [CID:5479539], cefoxitin [CID:441199], ertapenem [CID:150610], tigecycline [CID:54686904] and piperacillin/tazobactam [CID:23669315] were retrieved from Pubchem compound database [13]. Lamarckian genetic algorithm [14] was used to generate possible protein-ligand binding conformations.

Molecular dynamics and simulation for docked complexes (high and low affinity alone) were performed using the Gromacs 4.5.5 software [15]. The ligand parameters were analysed using PRODRG server [16] in the framework of the GROMOS force field 43a1 [17], [18].

## Results

**Figure 1:**
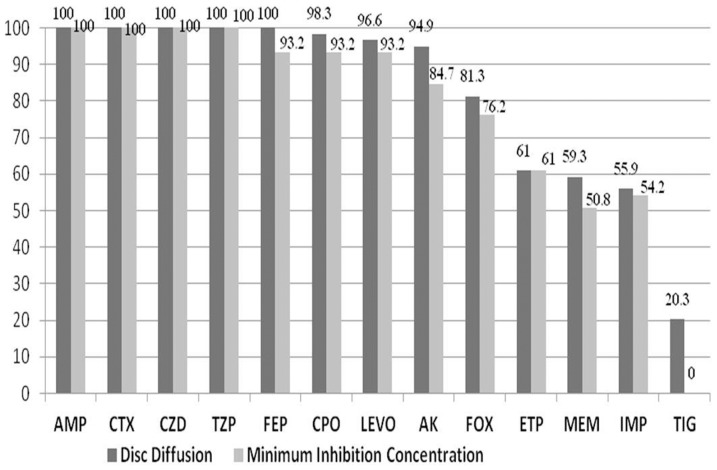
Antibiotic resistance pattern by Kirby Bauer method Vs E-test (MIC) (n = 59). Ampicillin (AMP), Cefotaxime (CTX), Ceftazidime (CZD), Piperacillin/Tazobactam (TZP), Cefepime (FEP), Cefpirome (CPO), Levofloxacin (LEVO), Amikacin (AK), Cefoxitin (FOX), Ertapenem (ETP), Meropenem (MEM), Imipenem (IMP), and Tigecycline (TIG).

Overall, 151 isolates were analysed by Kirby Bauer disk diffusion method and resistance to ampicillin (98.6%), cefotaxime (84.7%), cefpirome (81.4%), ceftazidime (79.4%), cefepime (70.8%), levofloxacin (61.5%), amikacin (61.5%), cefoxitin (44.3%), piperacillin/tazobactam (39%), ertapenem (24.5%), meropenem (23.8%), imipenem (22.5%), and tigecycline (15.7%) noted (Table 1). In our present study of 151 *K. pneumoniae* strains, 39% (59) are found to be resistant to piperacillin/tazobactam (β-lactam/inhibitor complex). These strains were selected for further testing to determine the MIC by E-test and results compared with disk diffusion method (Figure 1). Among the 151 study isolates 122 (81%) were ESBL's and additionally 38 were carbapenem resistant. The Pearson correlation analysis showed there was a positive association between ESBL production and resistance to ampicillin, imipenem, ertapenem at p values of 0.003, 0.006 and 0.001 respectively, rest of the antibiotics had p values<0.001. Only in the case of tigecycline no statistically significant association (p value = 0.140) was observed between resistance and ESBL production.

**Table 1:** Comparison of binding energy and percentage of resistance.

S.No	Ligands	*bla*OXA-1	
		Binding energy (Kcal/Mol)	% of resistance (Kirby Bauer method)
1	Ampicillin	−5.59	100
2	Cefotaxime	−6.5	100
3	Ceftazidime	−6.34	100
4	Amikacin	−5.3	83.3
5	Levofloxacin	−6.29	91.6
6	Imipenem	−5.28	25
7	Meropenem	−4.65	25
8	Cefepime	−5.86	100
9	Cefpirome	−5.65	100
10	Cefoxitin	−3.06	66.6
11	Ertapenem	−6.32	25
12	Tigecycline	−1.99	25
13	Piperacillin/Tazobactam	−6.74	100

MIC was performed for 59 *K. pneumoniae* inhibitor resistant isolates, and they were found to be resistant to ampicillin (100%), ceftazidime (100%), and cefotaxime (100%), piperacillin/tazobactam (100%), cefepime (93.2%), cefpirome (93.2%), levofloxacin (93.2%), amikacin (84.7%), cefoxitin (76.2%), ertapenem (61%), imipenem (54.2%) and meropenem (50.8%), all were susceptible to tigecycline (Figure 1).

### Molecular Methods

**Figure 2:**
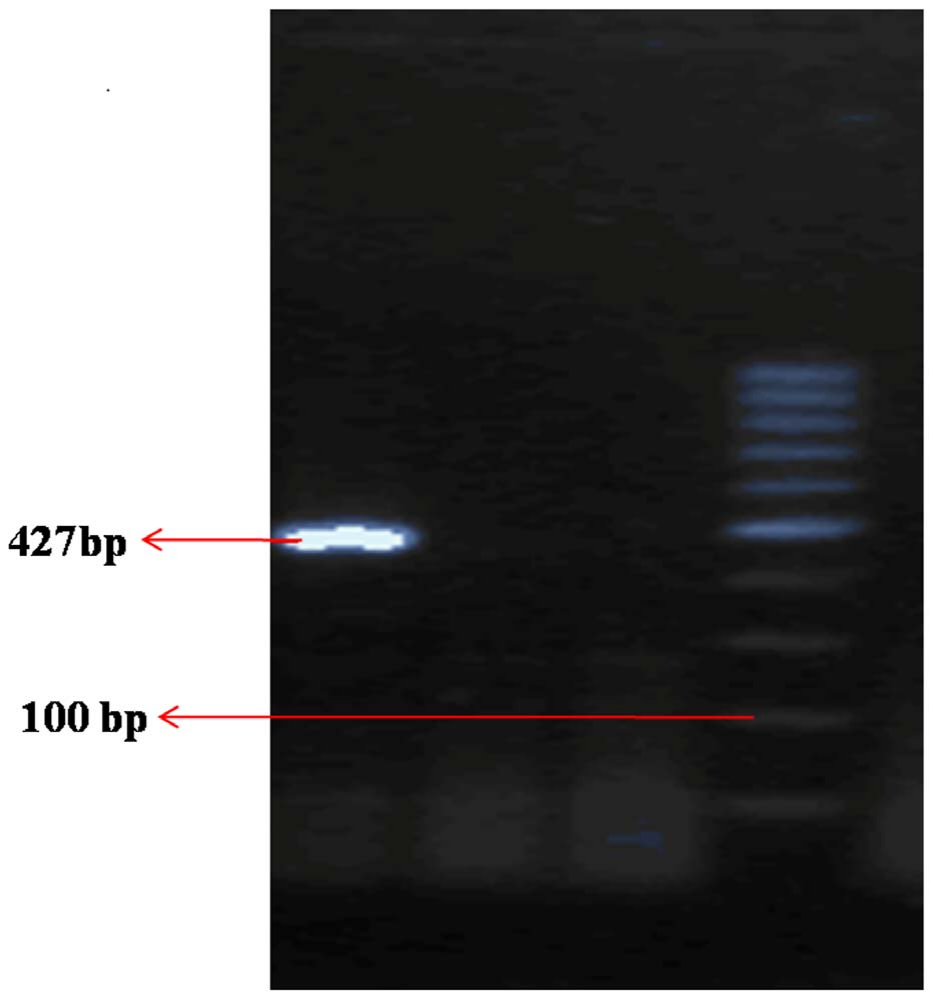
Gel picture of *bla*OXA-1. Lane 1: 3MB09 (Positive *bla*OXA-1 (427 bp), Lane 2: 3ADB28 (Negative), Lane 3: Negative control (sterile water), Lane 4 : Ladder 100 bp.

Phenotypically confirmed 59 piperacillin/tazobactam resistant isolates are used to detect *bla*OXA-1 gene by genotypic test. Amplification of β-lactamase genes shows the presence of *bla*OXA-1 on among 20.3% (12/59) study isolates (Figure 2).

### Bioinfomatics

#### Homology modeling

**Figure 3:**
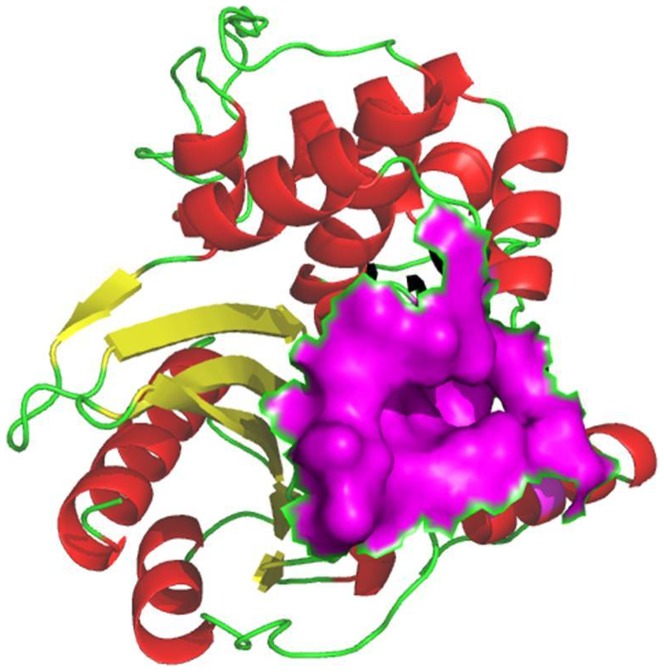
Final model structure for *bla*OXA-1 from *K. pneumoniae*. The α-helix is represented in red color, β-sheet by yellow arrows and loops by green lines. Acitve site is obtained by Q-siteFinder and shown in magenta color surface model.

The absence of the 3D-structure for *bla*OXA-1 from *K. pneumoniae* in PDB prompted us to construct the 3D-model of the protein of interest. The modelled strain was found to be suitable for *in-silico* analysis based on validation by Ramachandran Plot [11]. (Figure 3)

#### Active site prediction and Docking

The active site renders on the modelled protein was identified using Q-siteFinder. Among the ten sites obtained from Q-siteFinder, site 2 is conserved in *bla*OXA-1 and therefore chosen as binding site for docking study. The active site amino acids strings are ILE15, ALA16, ILE19, ALA49, SER50, THR51, ASN52, PHE142, ASP143, TYR144, GLY145, GLU174, GLN177, PHE178, ARG180, LYS181, ASN184, ASN186 and LEU187. From the description of Q-siteFinder it was observed that there was at least one successful prediction among the top three predicted site. The active residues are shown in surface model and represented in Figure 3. Docking of modelled *bla*OXA-1 protein was done with β-lactam antibiotics and piperacillin/tazobactam combination. Final docked conformations obtained for these compounds were evaluated based on the binding energy, number of hydrogen bonds formed and bond distances between atomic co-ordinates of the active site and ligand. The docking result of these compounds is given in table 2. All the 13 ligands accepted poses with the target proteins. The best binding conformation of each compound into binding site of each target protein were determined and the one having the lowest interaction energies of both docking energy among the 10 different poses were generated. Among the β-lactam antibiotics selected for this study, molecular docking analysis of ceftazidime shows the lowest binding energy value (−6.34 Kcal/Mol). On comparing the binding energy of all the complexes, tigecycline is shown to have least binding affinity with the *bla*OXA-1 (−1.99 Kcal/Mol). We further analyzed the docked conformation for finding the binding mode of ceftazidime and tigecycline into selected target proteins to validate the position obtained most likely to represent reasonable binding modes or conformations.

**Table 2:** Docking results for *bla*OXA-1.

S.No	Ligands	Pubchem Compound ID	*bla*OXA-1	
			Binding energy (Kcal/Mol)	No of H-bonds
1	Ampicillin	CID : 6249	−5.59	5
2	Cefotaxime	CID : 5742673	−6.5	9
3	Ceftazidime	CID : 481173	−6.34	9
4	Amikacin	CID : 37768	−5.3	13
5	Levofloxacin	CID : 149096	−6.29	7
6	Imipenem	CID : 104838	−5.28	7
7	Meropenem	CID : 441130	−4.65	5
8	Cefepime	CID : 5479537	−5.86	7
9	Cefpirome	CID : 5479539	−5.65	8
10	Cefoxitin	CID : 441199	−3.06	4
11	Ertapenem	CID : 150610	−6.32	5
12	Tigecycline	CID : 54686904	−1.99	3
13	Piperacillin/Tazobactam	CID : 23669315	−6.74	8

#### Binding mode of Ceftazidime and Tigecycline in to model protein *bla*OXA-1

**Figure 4:**
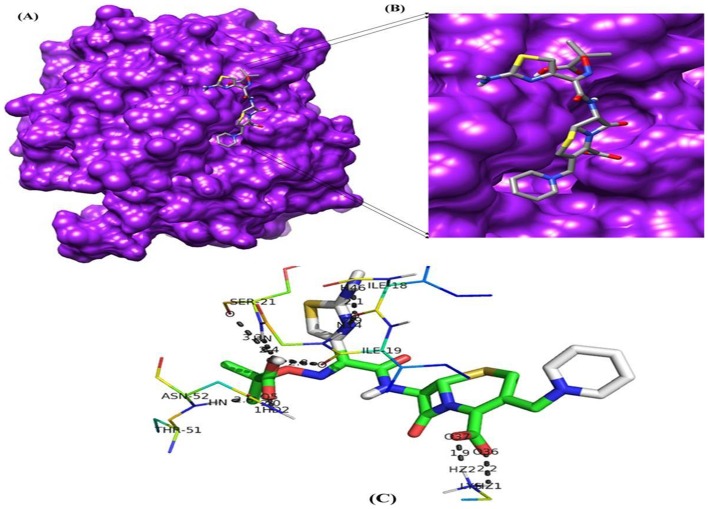
Molecular docking result of ceftazidime. (A) Binding pose of ceftazidime in the binding site of *bla*OXA-1 (B) A close-up view of the binding pose of ceftazidime; Protein structure is shown in surface model and the ligand is shown in stick model. (C) H-bond network with protein residues are shown.

**Figure 5:**
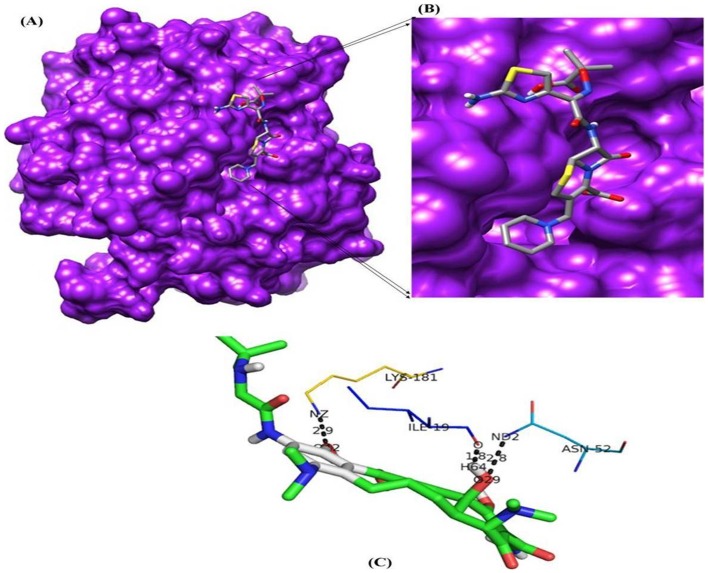
Molecular docking result of tigecycline. (A) Binding pose of tigecycline in the binding site of *bla*OXA-1 (B) A close-up view of the binding pose of tigecycline; Protein structure is shown in surface model and the ligand is shown in stick model. (C) H-bond network with protein residues are shown.

Binding mode of ceftazidime and tigecycline in model protein *bla*OXA-1 was analyzed. Ceftazidime resulted in the formation of 9 hydrogen bonds with the bond distance range of 1.9–3.3 A° and it was observed that side chain hydrogen atom of Lys181, Asn52, Ser21, Ile19 and Ile18 acts as hydrogen bond donor to interact with oxygen atom of the drug (Table 3). The best possible binding mode of ceftazidime in the *bla*OXA-1 binding site and corresponding 2D interaction models, hydrogen bonds and bond distance are shown in Figure 4. Docking simulation of tigecycline into model protein *bla*OXA-1 of *K. pneumoniae* reveals weak binding as indicated by formation of fewer hydrogen bonds (3). The binding energy estimated by autodock was −1.99 Kcal/Mol, which is higher binding energy value when compare to standard drug ceftazidime. It indicates tigecycline has lower binding affinity with modeled protein *bla*OXA-1 (Table 2, 3). From the interaction residues analysis it is observed that only three residues Lys181, Ile19 and Asn52 contributed H-bond interaction with *bla*OXA-1 (Figure 5). The comparative table with MIC results and binding energies are provided in Table 4.

**Table 3:** H-bond interaction and bond length obtained for Tigecycline and Ceftazidime with *bla*OXA-1.

Protein-ligand complex	H-bond interactions	Bond length (Å)
Tigecycline-*bla*OXA-1	(LYS181)N-O42	2.9
	(ILE19)O-H64	1.8
	(ASN52)N-O29	2.8
Ceftazidime-*bla*OXA-1	(LYS181)H-O36	2.2
	(LYS181)H-O37	1.9
	(ASNN52)HO-O5	2.0
	(ASN52)HNO-O5	2.0
	(SER21)HN-O6	2.4
	(SER21)O-O6	3.3
	(ILE19)O-O6	2.8
	(ILE18)O-N14	2.9
	(ILE18)O-H46	2.1

**Table 4:** Comparison of binding energy and percentage of resistance (MIC).

S.No	Ligands	*bla*OXA-1	
		Binding energy (Kcal/Mol)	% of resistance (MIC)
1	Ampicillin	−5.59	100
2	Cefotaxime	−6.5	100
3	Ceftazidime	−6.34	100
4	Amikacin	−5.3	75
5	Levofloxacin	−6.29	91.6
6	Imipenem	−5.28	16.6
7	Meropenem	−4.65	16.6
8	Cefepime	−5.86	100
9	Cefpirome	−5.65	100
10	Cefoxitin	−3.06	50
11	Ertapenem	−6.32	25
12	Tigecycline	−1.99	0
13	Piperacillin/Tazobactam	−6.74	100

#### MD simulation

**Figure 6:**
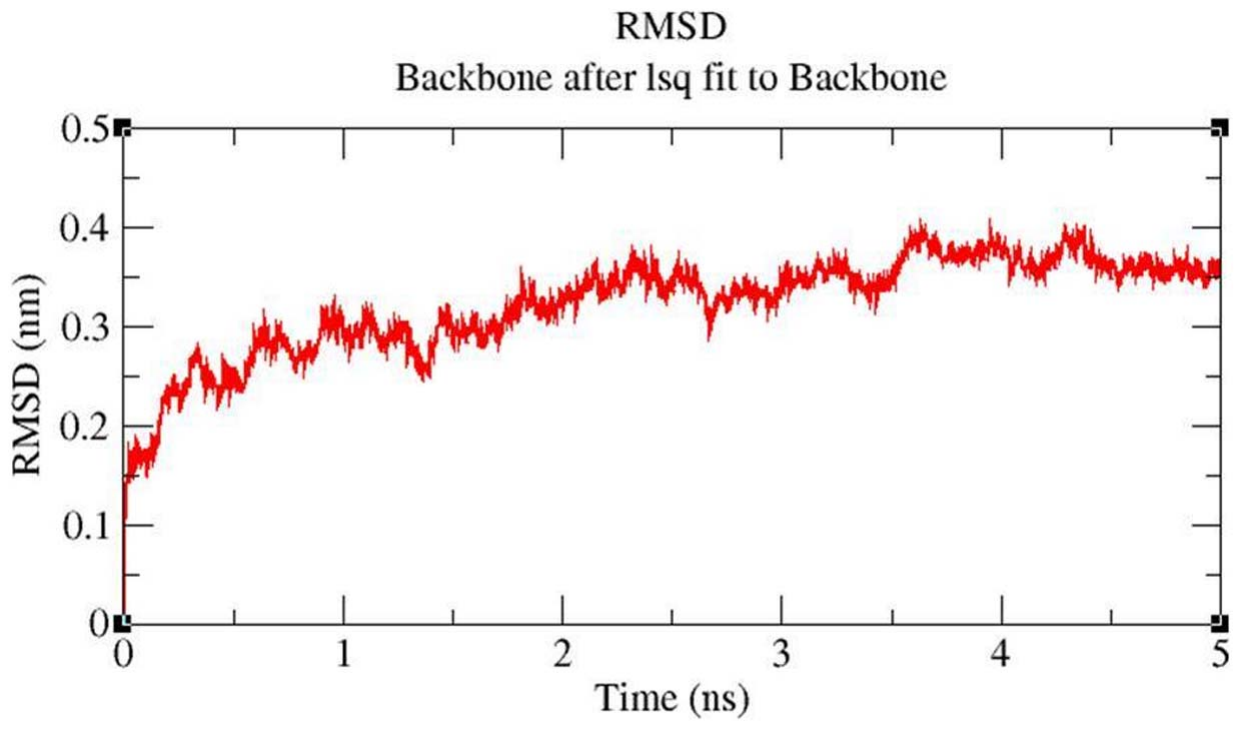
Backbone RMSDs are shown for tigecycline-*bla*OXA1 complex at 300K.

**Figure 7:**
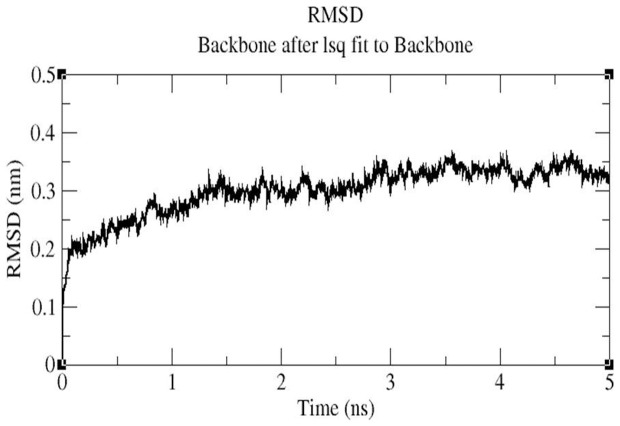
Backbone RMSDs are shown for ceftazidime-*bla*OXA-1 complex at 300K.

**Figure 8:**
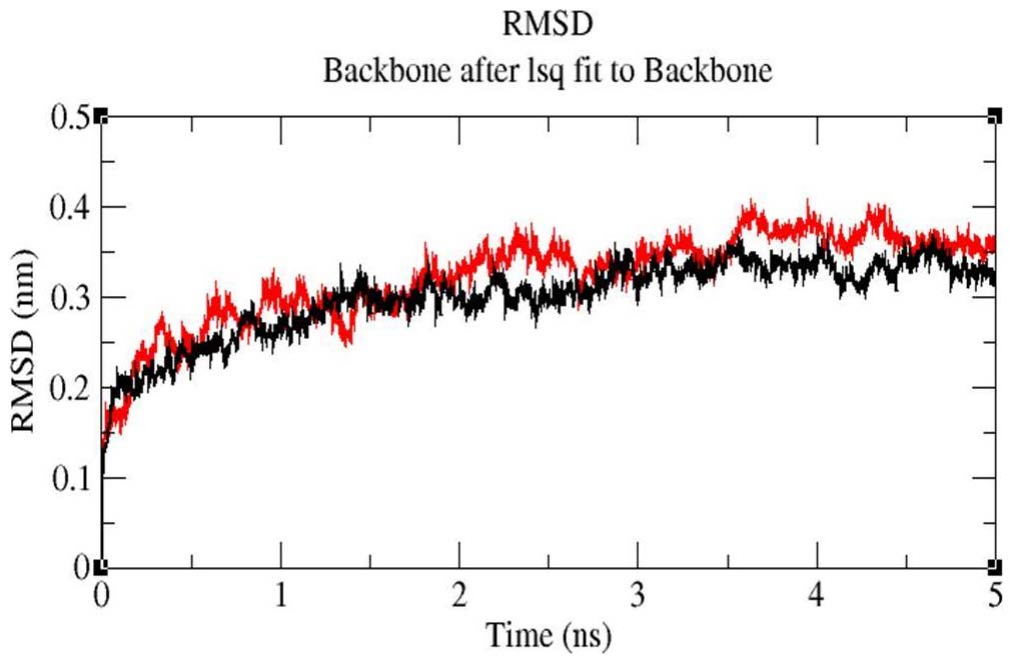
Backbone RMSDs are shown for protein-ligand complex at 300K, black color indicate RMSD for ceftazidime-*bla*OXA-1 complex, RMSD for tigecycline-*bla*OXA-1 complex shown in red.

MD simulations were carried out to explore the structural stabilities and protein internal motions within nanosecond (ns) time scale for ceftazidime-*bla*OXA-1 and tigecycline-*bla*OXA-1 complexes. The ceftazidime-*bla*OXA-1 and tigecycline-*bla*OXA-1 complexes were selected because ceftazidime has maximum binding energy (−6.34 Kcal/Mol) and tigecycline has least binding energy (−1.99 Kcal/Mol) with *bla*OXA-1. Each system was subjected to 5 ns MD simulations, and the results were analyzed to explore the stabilities and conformational changes of each complex. The root-mean-square-deviation (RMSD), a crucial parameter to analyse the equilibration of MD trajectories, were estimated by using the ceftazidime-*bla*OXA-1 and tigecycline-*bla*OXA-1 complex. Figure 6 shows the RMSD of ceftazidime relative to *bla*OXA-1. This system approaches equilibrium at 5 ns and was stable throughout the MD simulation, indicating a stable binding of ceftazidime with *bla*OXA-1. Figure 7 shows that the RMSD of tigecycline-*bla*OXA-1 complex which increases within the first 2 ns, the complex was unstable throughout the simulation. Interaction between tigecycline and *bla*OXA-1 is weak throughout the simulation. The comparison of RMSD value of ceftazidime-*bla*OXA-1 and tigecycline *bla*OXA-1 complexes are shown in Figure 8. RMSD values of tigecycline *bla*OXA-1 complex shows slight deviation when compared RMSD value of ceftazidime-*bla*OXA-1complex. The deviations described above indicate that how far the protein has moved from the native structure. It clearly indicates the interactions between tigecycline and *bla*OXA-1 is unstable throughout the simulation.

## Discussion

From our surveillance studies over the last 5 years, among 2782 Enterobacteriaceae strains, 285 isolates were carbapenem resistant. On analyzing a subset of these isolates (n = 86), a majority of them were found to carry ESBLs, Amp C and NDM enzymes mediating resistance. A few isolates were carbapenem resistant but negative for NDM, KPC, IMP and VIM, we therefore hypothesize that these isolates carry OXA mediated carbapenamase types and wished to explore this further (Manoharan A 2013-unpublished data). We believe OXA-1 co carriage along with ESBLs results in high levels of multidrug resistance including carbapenem resistance and proceeded to study this further. Further, given the high proportion of ESBLs which are screened and confirmed by the CLSI approved method of using clavulanic acid as an inhibitor we wished to evaluate tazobactam as an marker since this has been shown to possess a higher degree of [19] inhibitory activity against ESBLs as compared to clavulanic acid. Therefore we hypothesized that strains resistant to β-lactam plus clavulanic acid and β-lactam plus tazobactam would show high levels of multidrug resistance and proceeded to screen them for OXA-1 mediated resistance.

The prevalence of *bla*OXA-1-mediated resistance in India is not known, due to the limited number of surveillance studies seeking clinical strains producing OXA-1 β-lactamases and the difficulty that clinical microbiology laboratories have in accurately detecting this resistance mechanism. The present study showed phenotypic frequency of *bla*OXA-1 among *K.pneumoniae* in Indian medical centers is 39%, with commonly reported genotype seen among 20.3% of them.

In a previous study, 17 of 18 isolates obtained in 1989 primarily from patients with a history of travel to the Middle East, Asia, and Africa reported to produce a β-lactamase with similar properties to *bla*OXA-1. Ampicillin resistance in 75% of the *S. flexneri* strains was associated with the presence of OXA- 1 type β-lactamase. Taken together, these findings suggest the probable occurrence of host preference for OXA-type β -lactamase in *S. flexneri* [20].

In a study conducted at Hong Kong and Shanghai the production of β-lactamase from 91 ampicillin-resistant *Shigella flexneri* strains, *bla*TEM-1-like and *bla*OXA-1 like enzymes were identified in 21 and 79% of the strains. All *bla*OXA-type isolates had a low level of resistance to ampicillin, with MIC values of 128 or 256 µg/ml. All *bla*OXA-type isolates were susceptible to cephalothin and mercury chloride but resistant to streptomycin, chloramphenicol, spectinomycin, and tetracycline. The MICs of ampicillin/sulbactam and amoxicillin/clavulanate for *bla*OXA-type isolates were in the resistant range. The results suggested that *bla*OXA-type isolates are not inhibited by β-lactam–β-lactamase inhibitor combinations [21].

Our results indicate that *bla*OXA-1 carrying strains of *K.pneumoniae* exhibited 100% susceptibility to tigecycline, 83.4% to imipenem, meropenem, and 75% to ertapenem. β-lactam and β-lactamase inhibitor resistance alongwith multi drug resistance was noted to be high among septicemic patients. Carbapenem resistance was found to be between 20 to 25% and it may be primarily due to the production of New Delhi Metallo-β-lactamase (NDM). Resistance to β-lactam and β-lactamase inhibitor is mediated through co-carriage of multiple enzymes necessitating alternative therapeutic approach.

In a study in Spain conducted on 51 amoxicillin-clavulanic acid-resistant strains tested by multiplex PCR, 45 isolates had *bla*TEM and only two have *bla*SHV genes. Only one isolate had a *bla*OXA-1 gene. The simultaneous presence of two β-lactamase genes was not detected in this population of *E.coli* isolates. Hyperproduction of cephalosporinase can be suspected in routine testing by the classical antibiogram but other methods must be used to differentiate the mechanisms involving plasmid-encoded amoxicillin-clavulanic acid. TEM and *bla*OXA-1 enzymes are the major plasmid borne β-lactamases implicated in amoxicillin–clavulanic acid resistance in *E.coli* [22].

In our present findings majority of *bla*OXA-1 producing strains were resistant to β-lactamase inhibitor (piperacillin/tazobactam) but all are susceptible to tigecycline. Conventional PCR and *in-silco* approaches are suitable tools for screening plasmid-mediated β-lactamases among *K. pneumoniae* strains. The 3D-structure provides valuable insight into molecular function and also enables the analyses of its interactions with suitable inhibitors. PROCHECK and ProSA results reveal that the quality of the *bla*OXA-1 model is good. On the basis of experimental data, molecular docking studies on β-lactam antibiotics reveal that ceftazidime has highest binding affinity with *bla*OXA-1 and tigecycline has least binding affinity with *bla*OXA-1. On comparing the number of hydrogen bonds, tigecycline formed less number of hydrogen bonds (3) with *bla*OXA-1. Further molecular docking results of ceftazidime and tigecycline was validated by molecular dynamics study and revealed that tigecycline-*bla*OXA-1 complex is unstable and hence the *bla*OXA-1 β-lactam may not be able to cleave tigecycline. From the molecular docking and molecular dynamics analysis, it is confirmed that *bla*OXA-1 showed strong binding affinity with ceftazidime, resulting in cleavage of the antibiotic which suggests the high efficiency of *bla*OXA-1 in cleaving the β-lactam antibiotics. On the other hand, the lowest binding affinity of tigecycline to *bla*OXA-1 clearly shows the low enzyme activity of *bla*OXA-1, which in turn correlates well with the fact that tigecycline, is a very potent antibiotic. The calculated binding energy values are in agreement with corresponding MIC values by E-test and disc diffusion results. Higher binding energy indicates that OXA-1 β-lactamase has relatively weak binding affinity to β-lactam antibiotics. Lower binding energy value indicates strong binding affinity and thus bind strongly to β-lactam antibiotics resulting in cleavage of the antibiotic. Our insilico results are comparable to the in-vitro MIC values and disc diffusion which are presented in Table 1 and 4. At the study institution, the initial empiric treatment for severe, life threatening infections (associated with multi-organ dysfunction, septic shock) caused by Gram-negative bacteria is either imipenem or meropenem.Once the culture and susceptibility reports are available, the most appropriate antibiotic based on spectrum of activity, toxicity and cost (‘de-escalation’) is chosen. The result of the present study re-enforce this approach. The present study documents the emergence of multidrug resistant strains with OXA-1 co carriage. Antibiotic therapy needs to be based on laboratory confirmation of susceptibility which will help prevent occurrence and spread of antibiotic resistant strains.
